# Impact of centre volume on adrenalectomy outcomes: European multicentre study based on EUROCRINE^®^ registry

**DOI:** 10.1093/bjsopen/zraf180

**Published:** 2026-02-10

**Authors:** Yiğit Türk, Aykut Özkılıç, Francesco Pennestri, Marco Raffaelli, Radu Mihai, Murat Özdemir, Özer Makay

**Affiliations:** Division of Endocrine Surgery, Department of General Surgery, Ege University Hospital, Izmir, Türkiye; Division of Endocrine Surgery, Department of General Surgery, Ege University Hospital, Izmir, Türkiye; UOC di Chirurgia Endocrina e Metabolica, Fondazione Policlinico Universitario Agostino Gemelli IRCCS, Rome, Italy; Centro di Ricerca in Chirurgia Delle Ghiandole Endocrine e Dell’Obesità (CREO), Università Cattolica del Sacro Cuore, Rome, Italy; UOC di Chirurgia Endocrina e Metabolica, Fondazione Policlinico Universitario Agostino Gemelli IRCCS, Rome, Italy; Centro di Ricerca in Chirurgia Delle Ghiandole Endocrine e Dell’Obesità (CREO), Università Cattolica del Sacro Cuore, Rome, Italy; Department of Endocrine Surgery, Churchill Cancer Centre, Oxford University Hospitals NHS Foundation Trust, Oxford, UK; Division of Endocrine Surgery, Department of General Surgery, Ege University Hospital, Izmir, Türkiye; Centre for Endocrine Surgery, Özel Sağlık Hospital, İzmir, Türkiye; School of Medicine, Aristotle University, Thessaloniki, Greece; Instituto Português De Oncologia De Coimbra Francisco Gentil, Department of Surgery, Coimbra, Portugal

**Keywords:** centralization, perioperative results, caseload–results relationship, endocrine surgery, hospital performance

## Abstract

**Background:**

The relationship between surgeon volume and patient outcomes in adrenalectomy is well established in the literature and clinical guidelines. However, evidence regarding the impact of centre volume on patient outcomes remains limited. This study aimed to evaluate the effect of centre volume on patient outcomes.

**Methods:**

This multicentre study analysed adrenalectomy procedures from the EUROCRINE^®^ registry (2015–2024). A volume threshold was determined using receiver operating characteristic curve analysis to predict high-grade complications (Clavien–Dindo grade ≥ III). Outcomes were compared between high- and low-volume centres, and multivariable logistic regression was used to identify independent predictors of complications and death.

**Results:**

A total of 6672 patients undergoing adrenalectomy from 99 centres across Europe were included. The optimal centre volume threshold was ≥ 36 adrenalectomies per year. Only seven centres (7%) met this threshold, accounting for 36.7% of all procedures. High-volume centres had significantly lower rates of high-grade complications (0.9 *versus* 2.9%; *P* < 0.001), conversion (2.3 *versus* 3.5%), and reoperation (0.7 *versus* 1.6%), and shorter hospital stays (median 2 *versus* 3 days). Multivariable analysis showed high-volume centre status to be independently protective against high-grade complications (odds ratio 0.39, 95% confidence interval 0.24 to 0.63; *P* < 0.001), but not postoperative mortality (odds ratio 0.69; *P* = 0.480). Functional benign tumours compared with malignant tumours (odds ratio 0.61, 0.39 to 0.93; *P* = 0.020) and minimally invasive surgery (odds ratio 0.21, 0.14 to 0.31; *P* < 0.001) were both associated with a significantly lower risk of high-grade complications.

**Conclusion:**

Centre adrenalectomy volume is a key determinant of high-grade complication risk following adrenalectomy. A threshold of ≥ 36 adrenalectomies per year identifies high-performing centres. These findings support centralization of adrenal surgery to optimize outcomes and standardize care across institutions.

## Teaser

This multicentre retrospective study analysed 6672 patients who underwent adrenalectomy from the EUROCRINE^®^ registry to assess the impact of annual centre volume on surgical outcomes. Receiver operating characteristic curve analysis identified ≥ 36 adrenalectomies per year as the optimal threshold, with high-volume centres showing significantly lower rates of high-grade complications. These findings support centralization of adrenal surgery to optimize outcomes and standardize care.

## Introduction

Adrenalectomy is performed for a wide range of indications, from small benign hormone-secreting tumours to complex malignant lesions. Advances in computed tomography have led to increased detection of incidental adrenal masses, subsequently increasing the frequency of adrenalectomy. Recent studies^1,2^ have shown that over 80% of surgeons undertake only one adrenalectomy per year. Similarly, a study^3^ from Japan reported that 80% of benign laparoscopic adrenalectomies were performed in centres undertaking fewer than five adrenalectomies annually.

The impact of surgeons’ annual workload of adrenalectomy on patient outcomes has been investigated in numerous studies, consistently demonstrating improved outcomes with increasing surgical volume^1,2,4,5^. A minimum workload of 6 procedures per year for surgeon volume threshold has been recommended by the European Society of Endocrine Surgeons^6^ for benign disease, and 12 per year for those offering surgery for phaeochromocytomas and adrenocortical cancer. Similar recommendations were made in the American Association of Endocrine Surgeons guidelines^5^.

It is widely accepted that surgical outcomes improve when procedures are performed by experienced surgeons operating in high-volume centres, particularly those with standardized perioperative protocols and multidisciplinary teams^7,8^. Recent large-scale studies^1,4,9,10^ have further confirmed that high-volume surgeons and centres are associated with markedly lower rates of complications and mortality. An analysis^11^ of over 4400 adrenalectomies performed in the UK showed that the incidence of complications, conversion rates, and length of hospital stay are lower in centres undertaking more than 12 procedures per year. However, no clear consensus exists regarding institutional volume thresholds. Recent studies have proposed higher benchmarks to define high-volume centres. Waseda *et al*.^3^ suggested a threshold of 20 procedures per year, Bergamini *et al*.^12^ proposed 30 per year, and Uttinger *et al*.^10^ identified 31 annual procedures as a meaningful cut-off associated with improved outcomes,.

Using data from the EUROCRINE^®^ quality registry, this study investigated the impact of centre case volume on surgical outcomes following adrenalectomy. Specifically, the study aimed to determine whether higher annual adrenalectomy volumes are associated with improved perioperative outcomes, and to establish a data-driven volume threshold that can inform future guidelines on centralization and quality improvement in adrenal surgery.

## Methods

### Data source and study population

Data were retrieved from EUROCRINE^®^, a multinational endocrine surgical quality registry established within the European Union to reduce morbidity and mortality rates in patients with endocrine tumours. Participating centres are granted access to the registry after their institutional governing body has signed the necessary agreement. Data entry is voluntary. Submitted data are used for quality control analyses at both institutional and aggregated national or supranational levels. The EUROCRINE board oversees data compliance at the national level and approves access to anonymized data to be used in research projects^13^.

Each centre adhered to its own clinical protocols, surgical indications, and technical preferences. The study received approval from the Ethics Committee of Ege University (approval number 23-11.1T/39) and the EUROCRINE^®^ Steering Committee.

### Patient and variables

Baseline patient characteristics comprised age, sex, adrenal mass laterality, body mass index (BMI), and tumour size. Surgical indications were classified as functioning tumours (primary hyperaldosteronism, Cushing’s syndrome, phaeochromocytoma, mild autonomous cortisol secretion (MACS), and androgen-secreting tumours), benign non-functioning tumours (excluding malignancy), and malignant tumours (ACC, malignant phaeochromocytoma, and metastasis) based on preoperative clinical diagnosis.

Surgical techniques were grouped into minimally invasive and conventional approaches. Minimally invasive techniques encompassed transabdominal and posterior endoscopic adrenalectomies, as well as transabdominal robotically assisted and posterior robotically assisted approaches. Conventional techniques included laparotomy, open retroperitoneal, and thoracoabdominal approaches.

The primary outcomes were postoperative complications and death, classified according to the Clavien–Dindo system. Grades I and II were considered low-grade complications, grades IIIa, IIIb, and IV were defined as high-grade complications, and grade V was classified as postoperative death^14^.

### Centre volume threshold determination

Average annual centre adrenalectomy volume was determined by dividing the total number of adrenalectomies by the number of active reporting years, defined as years with at least one recorded adrenalectomy from individual centres. Baseline demographic and clinical characteristics were then compared across centres. Statistically significant differences across centres (*P* < 0.050) were observed for multiple variables: age, BMI, tumour size, sex, laterality, surgical indication, complications, surgical technique, and postoperative death.

To further assess the association between institutional case volume and postoperative outcomes, a multivariable linear regression model was constructed, incorporating each centre’s annual adrenalectomy volume as a continuous variable. The analysis identified several independent predictors of surgical outcomes, including patient age, sex, tumour laterality, tumour size, surgical indication, severity of complications, and surgical technique. Variance inflation factor values remained below 5, confirming the absence of multicollinearity among co-variables.

Receiver operating characteristic (ROC) curve analysis was conducted to determine the optimal threshold for classifying high-volume centres based on the prediction of high-grade complications.

### Statistical analysis

The cut-off point for annual adrenalectomy volume was identified using the Youden index derived from the ROC curve. Patients were stratified into two categories based on the annual adrenalectomy volume of the centre: high-volume or low-volume. Categorical variables were compared using the χ^2^ test or Fisher’s exact test, as appropriate. The Mann–Whitney *U* test was employed for continuous variables. Multivariable logistic regression analysis was conducted to identify independent associations between centre volume and the occurrence of high-grade postoperative complications and death. *P* < 0.050 was considered statistically significant. Statistical analyses were conducted using SPSS^®^ Statistics for Windows^®^ version 25.0 (IBM, Armonk, NY, USA).

## Results

### Cohort characteristics

Between January 2015 and December 2024, a total of 7200 adrenal operations performed in 100 centres were recorded in the EUROCRINE^®^ database. Patients were excluded if data were missing for surgical indication (126, 1.7%), surgical approach (142, 1.9%), Clavien–Dindo classification (426, 5.9%), or laterality (23, 0.3%), or if they were aged less than 18 years (28, 0.4%). One centre was excluded because all of its patients had missing data after these exclusions had been applied (*Table S1*). A total of 6672 patients from 99 centres across 20 European countries were included in the final evaluation after excluding records with missing data (*Table S2*).

The median duration of data reporting per centre was 4 (range 1–10) years, with a median annual adrenalectomy volume of 6 (interquartile range (i.q.r.) 3–15, range 1–147). Notably, nine centres reported only one procedure per year.

Median patient age was 56 (i.q.r. 45–65) years, and 3875 of the patients (58.1%) were women. The laterality distribution comprised right-sided procedures in 44.8%, left-sided procedures in 51.9%, and bilateral procedures in 3.3%.

Surgery was undertaken for a functioning tumour in 4163 patients (62.4%), including primary hyperaldosteronism (1648), phaeochromocytoma (1378), Cushing’s syndrome (951), MACS (175), and androgen-secreting tumour (11). Malignant indications accounted for 1072 procedures (16.1%), comprising metastases (720), adrenocortical carcinoma (ACC) (307), and malignant phaeochromocytoma (45). Benign non-functioning tumours were operated on in of 1437 patients (21.5%).

Minimally invasive approaches were used in 5886 patients (88.2%), including transabdominal laparoscopic (3477), retroperitoneoscopic (1580), transabdominal robotically assisted (803), and posterior robotically assisted (26) procedures. Open surgery was performed in 786 patients (11.8%), comprising conventional laparotomy (703), and open retroperitoneal (32), and thoracoabdominal (51) approaches.

Postoperative complications were recorded in 1021 patients (15.3%). Low-grade complications (Clavien–Dindo I–II) occurred in 13.2%, high-grade complications (IIIa–IV) in 2.1%, and there were 24 in-hospital deaths (grade V) (0.4%).

### Centre volume threshold

**Fig. 1:**
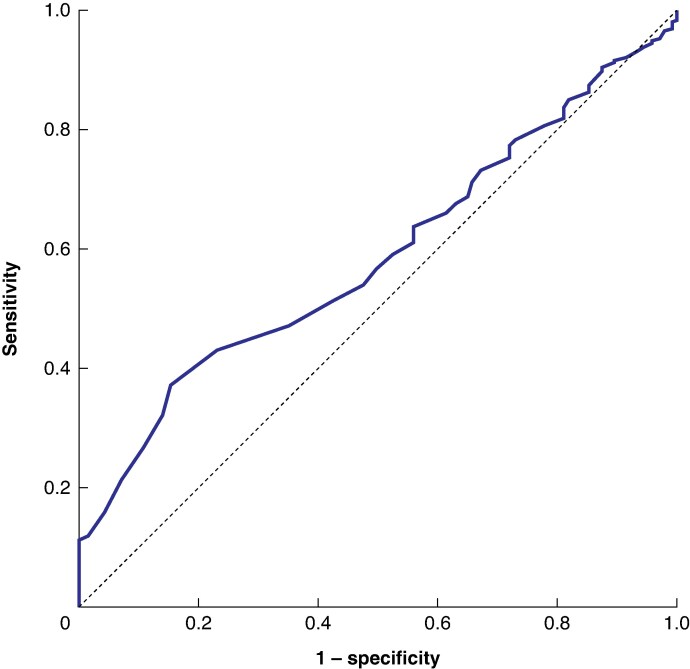
Receiver operating characteristic curve for the prediction of high-grade complications based on annual centre volume Area under the curve 0.586 (95% confidence interval 0.547 to 0.625; *P <* 0.001).

The optimal threshold for defining high-volume centres was determined based on the prediction of high-grade complications, using the Youden index derived from ROC curve analysis. This analysis identified a cut-off of 36 adrenalectomies per year (area under the curve (AUC) 0.586, 95% confidence interval (c.i.) 0.547 to 0.625; *P* < 0.001). At this threshold, the sensitivity was 37.2%, with a specificity of 84.7%, positive predictive value 5.4%, and negative predictive value 98.3%. These findings suggest that centres performing ≥ 36 adrenalectomies annually have a significantly lower likelihood of encountering high-grade complications (*Fig. 1*).

Applying this volume threshold, only 7 centres (7.1%) were categorized as high-volume institutions, yet they performed 2449 adrenalectomies, representing 36.7% of all procedures included in the study.

### Surgical indication

Functioning tumours were the leading indication in both cohorts, and there was no significant difference in prevalence between low- and high-volume centres (62.9 *versus* 61.5%; *P* = 0.25). However, benign non-functioning tumours were more prevalent in high-volume centres (26.3 *versus* 18.8%; *P* < 0.001), whereas malignant indications were more prevalent in low-volume centres (18.3 *versus* 12.2%; *P* < 0.001) (*Table 1*).

**Table 1:** Comparative analysis of demographics, surgical characteristics, and outcomes between low- and high-volume centres

	Low-volume centres (*n* = 4223)	High-volume centres (*n* = 2449)	P*
Age (years), median (i.q.r.)	56 (55–55)	56 (54–55)	0.300†
**Sex**			<0.001
Female	2321 (55.0%)	1554 (63.5%)	
Male	1902 (45.0%)	895 (36.5%)	
**Laterality**			
Right	1821 (43.1%)	1169 (47.7%)	<0.001
Left	2235 (52.9%)	1230 (50.2%)	0.030
Bilateral	167 (4.0%)	50 (2.0%)	<0.001
BMI (kg/m²), median (i.q.r.)	27 (24–31)	27 (24–31)	0.120†
Tumour size (mm), median (i.q.r.)	15 (6–35)	15 (5–35)	0.260†
**Surgical indication**			
Primary hyperaldosteronism	1169 (27.7%)	479 (19.6%)	<0.001
Excluding malignancy	793 (18.8%)	644 (26.3%)	<0.001
Phaeochromocytoma	865 (20.5%)	513 (20.9%)	0.670
Cushing’s syndrome	568 (13.5%)	383 (15.6%)	0.010
Metastasis	533 (12.6%)	187 (7.6%)	<0.001
Adrenocortical carcinoma	211 (5.0%)	96 (3.9%)	0.050
Mild autonomous cortisol secretion	46 (1.1%)	129 (5.3%)	<0.001
Malignant phaeochromocytoma	29 (0.7%)	16 (0.7%)	1.00
Androgen-secreting tumour	9 (0.2%)	2 (0.1%)	0.330
**Surgical indication—tumour type**			
Functioning tumour	2657 (62.9%)	1506 (61.5%)	0.250
Benign non-functioning tumour	793 (18.8%)	644 (26.3%)	<0.001
Malignant tumour	773 (18.3%)	299 (12.2%)	<0.001
**Operation type**			
Transabdominal endoscopic	2612 (61.9%)	865 (35.3%)	<0.001
Posterior endoscopic	790 (18.7%)	790 (32.3%)	<0.001
Transabdominal robotically assisted	188 (4.5%)	615 (25.1%)	<0.001
Laparotomy	543 (12.9%)	160 (6.5%)	<0.001
Thoracoabdominal approach	44 (1.0%)	7 (0.3%)	0.001
Open retroperitoneal	30 (0.7%)	2 (0.1%)	<0.001
Posterior robotically assisted	16 (0.4%)	10 (0.4%)	1.00
Minimally invasive surgery	3606 (85.4%)	2280 (93.1%)	<0.001
**Complication**			
Low-grade complication	406 (9.6%)	472 (19.3%)	<0.001
High-grade complication	121 (2.9%)	22 (0.9%)	<0.001
**Clavien–Dindo grade**			
I	230 (5.4%)	402 (16.4%)	<0.001
II	176 (4.1%)	70 (2.8%)	0.007
IIIa	48 (1.1%)	5 (0.2%)	<0.001
IIIb	48 (1.1%)	12 (0.4%)	0.007
IV	25 (0.5%)	5 (0.2%)	0.020
Postoperative death	19 (0.4%)	5 (0.2%)	0.130
**Resection margin status for malignant indication**			0.330
R1	88 (12.1%)	27 (9.9%)	
R2	19 (2.6%)	4 (1.5%)	
**Conversion to open surgery**	148 (3.5%)	58 (2.3%)	0.010
Malignant indications	66 (9.0%)	20 (7.1%)	0.340
Benign indications	82 (2.4%)	38 (1.8%)	0.130
Reoperation	65 (1.6%)	16 (0.7%)	0.002
Length of hospital stay (days), median (i.q.r.)	3 (2–5)	2 (1–4)	<0.001†

Values are *n* (%) unless otherwise stated. i.q.r., Interquartile range; BMI, body mass index. *χ^2^ test or Fisher’s exact test, except †Mann–Whitney *U* test.

Among functional tumour indications, primary hyperaldosteronism predominated and was significantly more frequent in low-volume centres (27.7 *versus* 19.6%; *P* < 0.001), whereas phaeochromocytoma occurred at similar rates (20.5 *versus* 20.9%; *P* = 0.670). Cushing’s syndrome was more prevalent in high-volume centres (15.6 *versus* 13.5%; *P* = 0.010). MACS was more prevalent in high-volume centres (5.3 *versus* 1.1%; *P* < 0.001), and androgen-secreting tumours were rare in both groups (0.1 *versus* 0.2% in high- and low-volume centres, respectively; *P* = 0.330).

Among malignant indications, metastasis was more frequently reported from low-volume centres (12.6 *versus* 7.6%; *P* < 0.001). The rate of ACC was borderline higher in low-volume centres (5.0 *versus* 3.9%; *P* = 0.050). The rates of malignant phaeochromocytoma were identical in both groups (0.7%; *P* = 1.00) (*Table 1*).

### Surgical techniques

Surgical approaches differed significantly between high- and low-volume centres. When categorized as minimally invasive *versus* open techniques, minimally invasive procedures were performed in 93.1% of patients at high-volume centres compared with 85.4% at low-volume centres (*P* < 0.001).

Laparoscopic transabdominal adrenalectomy was the most common procedure overall and was significantly more prevalent in low-volume centres (61.9 *versus* 35.3%; *P* < 0.001). High-volume centres more frequently employed posterior laparoscopic approaches (32.3 *versus* 18.7%; *P* < 0.001) and transabdominal robotically assisted techniques (25.1 *versus* 4.5%; *P*<<0.001). The prevalence of posterior robotically assisted adrenalectomy was identical in the two groups (0.4%; *P* = 1.00). Open surgery was more common in low-volume centres (12.9 *versus* 6.5%; *P* < 0.001), with thoracoabdominal access also slightly more frequent in these centres (1.0 *versus* 0.3%; *P* < 0.001) (*Table 1*).

### Postoperative complications

Assignment of Clavien–Dindo complication grades differed significantly between high- and low-volume centres. Low-grade complications were more frequently observed in high-volume centres (19.3 *versus* 9.6%; *P* < 0.001), whereas high-grade complications occurred more commonly in low-volume centres (2.9 *versus* 0.9%; *P* < 0.001). Grade I complications were more common in high-volume centres (16.4 *versus* 5.4%; *P* < 0.001). Although low-grade complications overall were more prevalent in high-volume centres, grade II complications occurred more frequently in low-volume centres (4.1 *versus* 2.8%; *P* = 0.007). All high-grade complications were less frequent in high-volume centres: grade IIIa (1.1 *versus* 0.2%; *P* < 0.001), grade IIIb (1.1 *versus* 0.4%; *P* = 0.007), and grade 4 (0.5 *versus* 0.2%; *P* = 0.020).

Laparoscopic-to-open conversion was less frequent in high-volume centres than low-volume centres (2.3 *versus* 3.5%; *P* = 0.010). Following stratification by indication, conversion rates did not differ significantly between low- and high-volume centres in procedures for either malignant (9.0 *versus* 7.1%; *P* = 0.340) or benign (2.4 *versus* 1.8%; *P* = 0.130) disease. Reoperation rates were significantly lower in high-volume centres (0.7 *versus* 1.6%; *P* = 0.002).

Median hospital stay was shorter in high-volume compared with low-volume centres: 2 (i.q.r. 1–4) *versus* 3 (2–5) days (*P* < 0.001).

In multivariable logistic regression analysis, a minimally invasive approach was independently associated with a significantly lower risk of high-grade complications (odds ratio (OR) 0.21, 95% c.i. 0.14 to 0.31; *P* < 0.001). Compared with malignant indications, functioning tumours were associated with a reduced risk (OR 0.61, 0.39 to 0.93; *P* = 0.02), whereas there was no statistical significance for benign non-functioning tumours (*P* = 0.150). Having surgery at a high-volume centre was also an independent protective factor (OR 0.39, 0.24 to 0.63; *P* < 0.001). Patient sex and tumour laterality were not significantly associated with high-grade complications in this model (*Table 2*).

**Table 2:** Multivariable logistic regression analysis of factors associated with high-grade complications and postoperative death

	High-grade complications		Postoperative death	
	Odds ratio	*P*	Odds ratio	*P*
Female sex	1.27 (0.91, 1.79)	0.15	0.49 (0.58, 3.02)	0.490
**Laterality**				
Right	1.00 (reference)		1.00 (reference)	
Left	1.29 (0.87, 1.76)	0.23	0.91 (0.38, 2.17)	0.840
Bilateral	2.01 (0.92, 4.34)	0.76	4.76 (1.24, 18.2)	0.020
Minimally invasive approach	0.21 (0.14, 0.31)	<0.001	0.13 (0.05, 0.34)	<0.001
**Surgical indication—tumour type**				
Malignant tumour	1.00 (reference)		1.00 (reference)	
Benign non-functioning tumour	0.70 (0.43, 1.14)	0.15	0.47 (0.16, 1.34)	0.150
Functioning tumour	0.61 (0.39, 0.93)	0.02	0.16 (0.05, 0.56)	0.004
High-volume centre	0.39 (0.24, 0.63)	<0.001	0.69 (0.25, 1.90)	0.480

Values in parentheses are 95% confidence intervals.

Among patients who developed severe postoperative complications (Clavien–Dindo grade ≥ IIIa), infection and bleeding requiring transfusion were the most frequent causes across all severity grades. Among grade IIIa complications (45 with specified causes), infection accounted for 42.2% and bleeding for 22.2%, followed by pneumothorax (20.0%) and less common events such as myocardial infarction, postoperative ileus, pancreatic leak, pancreatic fistula, stroke, cardiac arrhythmia, acute renal failure, diaphragmatic injury, peptic ulcer bleeding, anastomotic leak, and pneumonia. Among grade IIIb complications (75 with specified causes), bleeding was the predominant cause (26.7%), followed by infection (16.0%), pancreatic fistula (9.3%), anastomotic leak (5.3%), and ileus (4.0%), with others including pneumothorax, pulmonary embolism, fascial dehiscence, colonic injury, strangulated port-site hernia, and pneumonia. For grade IV complications (39 with specified causes), bleeding remained the leading cause (23.1%), followed by acute renal failure (10.3%), infection (7.7%), pancreatic fistula (7.7%), pneumonia (7.7%), and less frequent but serious events such as perforation, deep vein thrombosis, stroke, pulmonary embolism, liver laceration, and pneumothorax.

### Postoperative mortality

Postoperative death was rare in both groups but appeared to occur more frequently in low-volume centres (0.4 *versus* 0.2%; *P* = 0.130) (*Table 1*).

In multivariable logistic regression analysis, a minimally invasive approach was independently associated with a significantly lower risk of postoperative death (OR 0.13, 95% c.i. 0.05 to 0.34; *P* < 0.001). Compared with malignant indications, functioning tumours were associated with a markedly reduced risk (OR 0.16, 0.05 to 0.56; *P* = 0.004), whereas there was no statistical significance for benign non-functioning tumours (*P* = 0.15). Bilateral adrenalectomy was independently linked to an increased risk of postoperative death (OR 4.76, 1.24 to 18.20; *P* = 0.020). In this model, patient sex, laterality, and surgery at a high-volume centre showed no significant association with mortality (*Table 2*).

## Discussion

This international, multicentre retrospective study of 6672 adrenalectomy procedures recorded in the EUROCRINE^®^ registry across 99 European centres has demonstrated that institutional adrenalectomy volume is a strong and independent predictor of high-grade postoperative complications. Using ROC curve analysis, an optimal centre volume threshold of 36 adrenalectomies per year was identified. Centres performing above this threshold showed significantly lower rates of high-grade complications, conversion to open surgery, and reoperation, and had shorter hospital stays. However, the difference in postoperative mortality was not statistically significant. These findings support the extension of the volume–outcome relationship to adrenalectomy, highlighting that institutional experience may be just as critical as individual-surgeon experience. These results support the centralization of adrenal surgery to high-volume centres, where multidisciplinary teams and use of standardized protocols can optimize patient safety.

These results are consistent with the findings of Anderson *et al*.^1^, who used restricted cubic spline modelling to define a statistically valid volume–outcome relationship in adrenal surgery. In contrast, previous studies^2,4,9–11,15,16^ relied on subjective thresholds or stratified volume groups (for example quartiles, tertiles or previous results). In the present study, ROC curve analysis was used not to design a highly discriminative predictive model, but to determine an outcome-based threshold that could identify high-volume centres. Although the AUC value of 0.586 indicated modest discriminative power, it provided a statistically valid cut-off associated with a lower risk of high-grade complications. This cut-off remained significant in both univariable and multivariable analyses, thereby supporting an evidence-based definition of high-volume centres in adrenal surgery.

Adrenal surgery is relatively rare, and this low frequency is reflected in surgical practice patterns worldwide. Anderson *et al*.^1^ reported that 82.3% of surgeons performed only one adrenalectomy annually, and these surgeons had the highest complication rates. Similarly, Palazzo *et al*.^2^ found that 84% of surgeons in the UK undertook just one adrenal procedure per year. In Japan, Waseda *et al*.^3^ demonstrated that 80% of benign laparoscopic adrenalectomies were performed in centres conducting fewer than five such procedures annually. The present study has confirmed this pattern: although only 7 of the 99 centres carried out ≥ 36 adrenalectomies annually, these high-volume centres accounted for more than one-third of all procedures (36.7%), highlighting the concentrated nature of adrenal surgery in specialized institutions.

Several previous studies^1,2,4,11^ have reported that surgical volume is a key determinant of outcomes in adrenalectomy, with higher-volume surgeons achieving lower complication rates. Some studies^17^ have suggested that both surgeon and centre experience influence outcomes, with surgeon volume having a more pronounced effect. However, the well documented ‘centre effect’—reflecting the impact of multidisciplinary teams, standardized perioperative protocols, and institutional infrastructure—may also play a substantial role in adrenal surgery. This effect has been well established in other complex procedures such as gastrectomy, colectomy, lung resection, and pancreatic surgery^7,8^. Moreover, it has been demonstrated that high-volume centres with a multidisciplinary approach have a positive impact on the oncological outcome of patients with ACC, resulting in a lower recurrence rate and improved mean time to recurrence^18^.

In the present study, surgeon-specific volume data were not available in the database, which precluded separate assessment of surgeon and centre effects. However, at least theoretically, experience of the single surgeon in a high-volume centre could have a limited role because of standardization of the surgical technique and the possibility that less experienced surgeons can be tutored, proctored, and supervised by more experienced ones. That can ultimately result in a homogeneous outcome for different levels of experience^19^.

Multiple large-scale studies have highlighted the association between centre volume and clinical outcomes in adrenal surgery. Waseda *et al*.^3^ reported that centres performing 20 or more laparoscopic adrenalectomies annually had a complication rate of 7.2%, compared with 11.2% in those undertaking fewer than 5 procedures per year. Similarly, Park *et al*.^15^ documented complication rates of 16.3% in high-volume centres *versus* 24.5% in low-volume centres. In Germany, Uttinger *et al*.^10^ reported lower complication rates in centres performing more than 31 adrenalectomies annually (17.3 *versus* 23.1%). Bergamini *et al*.^12^ also noted a markedly reduced complication rate of 4.8% in reference centres performing over 30 procedures per year, compared with 22.0% in lower-volume centres. In contrast, Murphy *et al*.^20^ and Stavrakis *et al*.^16^ did not identify a statistically significant relationship between hospital volume and complication rates. In the present study, high-grade complications occurred in 0.9% of patients who had surgery in high-volume centres, compared with 2.9% at low-volume centres. Furthermore, in multivariable logistic regression analysis, having surgery at a high-volume centre was an independent protective factor against high-grade complications (OR 0.39, 95% c.i. 0.24 to 0.63; *P* < 0.001). These findings are consistent with those of previous studies and support the reliability of the cut-off value used to define high-volume centres in the present analysis.

Regarding postoperative mortality, previous studies reported inconsistent findings in relation to centre volume. Park *et al*.^15^, Uttinger *et al*.^10^, and Waseda *et al*.^3^ reported no significant differences in mortality rates between high- and low-volume centres. In contrast, Caiazzo *et al*.^9^ reported a significantly lower mortality rate of 0.9% in centres performing ≥ 32 adrenalectomies annually, compared with 1.8% in lower-volume institutions. In the present study, univariable analysis demonstrated a higher postoperative mortality rate in low-volume centres than in high-volume centres (0.4 *versus* 0.2%), although this difference did not reach statistical significance. Similarly, in multivariable logistic regression analysis, high-volume centre status was not independently associated with reduced mortality (OR 0.69; *P* = 0.48).

This study found that malignant indications were relatively more prevalent in low-volume centres. This may have been due to regional differences in referral practices, variations in case complexity, and differences in preoperative recording practices, with some patients with malignant disease possibly being managed locally. Such factors should be considered when interpreting this finding.

Low-grade complications were more prevalent in high-volume centres (19.3 *versus* 9.6%). Within the Clavien–Dindo classification, only grade I complications were more common in high-volume centres (16.4 *versus* 5.4%), whereas grades II, IIIa, IIIb, and IV were more frequent in low-volume centres. This difference may reflect more systematic coding and follow-up processes in high-volume centres, a lower threshold for recording minor deviations from the expected postoperative course, and the presence of comprehensive multidisciplinary teams that are more likely to detect and document such events.

The present data showed that high-volume centres more frequently employed minimally invasive techniques (93.1 *versus* 85.4%), particularly posterior and robotic approaches, which may have contributed to the observed improvements in surgical outcomes. Minimally invasive surgery was independently associated with a significantly reduced risk of both high-grade complications (OR 0.21) and postoperative death (OR 0.13). However, this association may partly reflect selection bias, as minimally invasive approaches are more likely to be used in patients with smaller tumours and benign indications. In the multivariable analysis, bilateral adrenalectomy emerged as an independent predictor of postoperative death (OR 4.76; *P* = 0.02), but was not significantly associated with high-grade complications (OR 2.01; *P* = 0.76). Malignant indication was independently linked to high-grade complications and postoperative death.

This study has several limitations. The retrospective design and reliance on registry-based data may have introduced reporting and selection biases. The database does not provide surgeon-specific procedure numbers, so it was not possible to adjust for surgeon volume. Variability in individual-surgeon’s experience and caseload in high-volume centres may therefore have influenced outcomes. Although the registry offers a large multicentre data set from across Europe, the findings may not be generalizable to healthcare systems with different organizational structures. Unmeasured confounders, such as variability in case complexity, perioperative care, and institutional protocols, may also have affected the results.

## Supplementary Material

zraf180_Supplementary_Data

## Data Availability

The data underlying this article were provided by the EUROCRINE^®^ registry with permission. Data will be shared on request to the corresponding author with the permission of the EUROCRINE^®^ board.
