# Supplementary material for: Impact of centre volume on adrenalectomy outcomes: European multicentre study based on EUROCRINE® registry
Source: BJS Open. 2026 Feb 10;10(1):zraf180. doi: 10.1093/bjsopen/zraf180 (PMC12888810; doi:10.1093/bjsopen/zraf180)
Supplement: zraf180_Supplementary_Data [file zraf180_supplementary_data.docx]

**Title: Impact of Centre Volume on Adrenalectomy Outcomes: European Multicentre Study based on EUROCRINE® Registry**

**Authors:** Yiğit Türk^1^, Aykut Özkılıç^1^, Francesco Pennestri^2,3^, Marco Raffaelli^2,3^, Radu Mihai^4^, Murat Özdemir^1^, Özer Makay^5,6,7^

^1^Affiliation: Division of Endocrine Surgery, Department of General Surgery, Ege University Hospital, 35100, Izmir, Türkiye

^2^Affiliation: UOC di Chirurgia Endocrina e Metabolica, Fondazione Policlinico Universitario Agostino Gemelli IRCCS, 00168 Rome, Italy

^3^Affiliation: Centro di Ricerca in Chirurgia delle Ghiandole Endocrine e dell'Obesità (C.R.E.O.), Università Cattolica del Sacro Cuore, Rome, Italy

^4^Affiliation: Department of Endocrine Surgery, Churchill Cancer Centre, Oxford University Hospitals NHS Foundation Trust, Oxford OX3 9DU, UK

^5^Affiliation: Özel Sağlık Hospital, Centre for Endocrine Surgery, İzmir, Türkiye

^6^Affiliation: Aristotle University, School of Medicine, Thessaloniki, Greece

^7^Affiliation: Instituto Português De Oncologia De Coimbra Francisco Gentil, Coimbra, Portugal

**Corresponding author:** Murat Özdemir, Associate Professor, MD

Address: Ankara street, Ege University Hospital, Department of General Surgery, Division of Endocrine Surgery,35100, Bornova, Izmir, Turkey

E-mail: muratozdemir.md@gmail.com Phone: +90 232 390 50 50

**Supplementary Materials - Index**

| **Supplementary Methods** |  |
| --- | --- |
| No supplementary methods were included. | *pag. 2* |
|  |  |
| **Supplementary Results** |  |
| No supplementary results were included. | *pag. 3* |
|  |  |
| **Supplementary Appendixes** |  |
| No supplementary appendixes were included. | *pag. 4* |
|  |  |
| **Supplementary Figures and Tables** |  |
| Supplementary Table S1. Missing data distribution by variables | *pag. 5* |
| Supplementary Table S2. Summary of included centres with annual case volume, complications, and mortality | *pag. 6-8* |
| **References** | *pag. 9* |
| None |  |

**Supplementary Methods**

No additional methods beyond those described in the main manuscript.

**Supplementary Results**

No additional results beyond those presented in the main manuscript.

**Supplementary Appendixes**

No supplementary appendixes were included.

**Supplementary Figures and Tables**

**Table S1.** Missing data distribution by variables

| *Variable* | *Missing data (n/%)* |
| --- | --- |
| Age | 0 (0%) |
| Sex | 0 (0%) |
| Laterality | 23 (0.32%) |
| Centres experiences | 0 (0%) |
| Surgical indications | 126 (1.75%) |
| Surgical approach | 142 (1.97%) |
| Complications and mortality | 426 (5.92%) |

**Table S2:** Summary of included centres with annual case volume (cases/year), complications (n), and mortality (n).

| Centre No | Annual Case Volume (Cases/year*) | Complication (*n*) | | Mortality (*n*) |
| --- | --- | --- | --- | --- |
|  |  | Low-grade | High grade |  |
| 1 | 4.3 | 1 | 0 | 0 |
| 2 | 147 | 0 | 0 | 0 |
| 3 | 40.6 | 39 | 5 | 1 |
| 4 | 15.2 | 9 | 1 | 0 |
| 5 | 4.5 | 2 | 0 | 0 |
| 6 | 31.4 | 14 | 10 | 1 |
| 7 | 5 | 1 | 0 | 0 |
| 8 | 8.7 | 7 | 1 | 0 |
| 9 | 7 | 1 | 0 | 0 |
| 10 | 14.3 | 8 | 0 | 0 |
| 11 | 15.7 | 1 | 0 | 0 |
| 12 | 1 | 0 | 0 | 0 |
| 13 | 1 | 0 | 0 | 0 |
| 14 | 2 | 1 | 0 | 0 |
| 15 | 5 | 1 | 0 | 0 |
| 16 | 1 | 0 | 0 | 0 |
| 17 | 14 | 3 | 0 | 0 |
| 18 | 7 | 2 | 0 | 0 |
| 19 | 29 | 3 | 1 | 0 |
| 20 | 6.7 | 1 | 0 | 0 |
| 21 | 14 | 0 | 0 | 0 |
| 22 | 38.4 | 320 | 5 | 0 |
| 23 | 2 | 0 | 0 | 0 |
| 24 | 10.6 | 11 | 1 | 0 |
| 25 | 6.8 | 13 | 3 | 0 |
| 26 | 5.7 | 0 | 1 | 0 |
| 27 | 9 | 8 | 0 | 0 |
| 28 | 1 | 0 | 0 | 0 |
| 29 | 2 | 0 | 0 | 0 |
| 30 | 16.2 | 7 | 3 | 0 |
| 31 | 1 | 0 | 0 | 0 |
| 32 | 4.5 | 0 | 0 | 0 |
| 33 | 3 | 0 | 0 | 0 |
| 34 | 28.6 | 11 | 7 | 0 |
| 35 | 1 | 0 | 0 | 0 |
| 36 | 43.7 | 11 | 4 | 0 |
| 37 | 20.6 | 14 | 4 | 1 |
| 38 | 21.3 | 12 | 3 | 0 |
| 39 | 34.8 | 26 | 10 | 0 |
| 40 | 11.1 | 11 | 0 | 0 |
| 41 | 3 | 3 | 0 | 0 |
| 42 | 3.9 | 5 | 0 | 0 |
| 43 | 1.5 | 1 | 0 | 0 |
| 44 | 6.6 | 19 | 3 | 0 |
| 45 | 1 | 0 | 0 | 0 |
| 46 | 53.6 | 57 | 4 | 0 |
| 47 | 62 | 12 | 2 | 0 |
| 48 | 14 | 0 | 1 | 0 |
| 49 | 1.5 | 0 | 0 | 0 |
| 50 | 1.3 | 2 | 0 | 0 |
| 51 | 2 | 0 | 0 | 0 |
| 52 | 1 | 0 | 0 | 0 |
| 53 | 2.5 | 0 | 0 | 0 |
| 54 | 34.8 | 7 | 1 | 0 |
| 55 | 4.7 | 1 | 2 | 0 |
| 56 | 31.8 | 9 | 7 | 2 |
| 57 | 3.6 | 3 | 0 | 1 |
| 58 | 4.5 | 1 | 0 | 0 |
| 59 | 24.7 | 7 | 10 | 5 |
| 60 | 15.5 | 9 | 1 | 1 |
| 61 | 14.4 | 14 | 7 | 1 |
| 62 | 18.1 | 9 | 0 | 1 |
| 63 | 9 | 6 | 0 | 0 |
| 64 | 4.2 | 3 | 1 | 0 |
| 65 | 6.2 | 7 | 0 | 0 |
| 66 | 4.5 | 10 | 1 | 0 |
| 67 | 8 | 1 | 0 | 0 |
| 68 | 37.6 | 33 | 2 | 4 |
| 69 | 1 | 0 | 0 | 0 |
| 70 | 4 | 0 | 1 | 0 |
| 71 | 3 | 0 | 0 | 0 |
| 72 | 3.7 | 1 | 0 | 1 |
| 73 | 2.3 | 0 | 0 | 0 |
| 74 | 5 | 2 | 0 | 0 |
| 75 | 17 | 0 | 0 | 0 |
| 76 | 5.5 | 0 | 0 | 0 |
| 77 | 15 | 15 | 2 | 0 |
| 78 | 15 | 0 | 0 | 0 |
| 79 | 3 | 0 | 0 | 0 |
| 80 | 9.9 | 3 | 5 | 0 |
| 81 | 11.3 | 13 | 5 | 1 |
| 82 | 5.4 | 2 | 2 | 0 |
| 83 | 18.5 | 8 | 5 | 0 |
| 84 | 2.7 | 1 | 0 | 0 |
| 85 | 8.9 | 6 | 1 | 0 |
| 86 | 6.2 | 4 | 2 | 1 |
| 87 | 2.6 | 1 | 0 | 0 |
| 88 | 3.8 | 0 | 0 | 0 |
| 89 | 8.7 | 8 | 1 | 0 |
| 90 | 1 | 0 | 0 | 0 |
| 91 | 2.7 | 0 | 0 | 0 |
| 92 | 13.3 | 5 | 2 | 0 |
| 93 | 4 | 0 | 0 | 0 |
| 94 | 17 | 1 | 1 | 0 |
| 95 | 1 | 0 | 0 | 0 |
| 96 | 17.4 | 42 | 8 | 1 |
| 97 | 12.3 | 5 | 5 | 1 |
| 98 | 3.1 | 1 | 1 | 0 |
| 99 | 5.5 | 3 | 1 | 1 |

*Annual case volume was calculated by dividing the total number of adrenalectomy cases contributed by each centre by the number of active reporting years in the EUROCRINE® registry.

**References**

None
